# Editorial: Reducing consumption of animal products

**DOI:** 10.3389/fnut.2023.1249873

**Published:** 2023-08-28

**Authors:** Christopher Bryant, Christopher J. Hopwood, Jared Piazza

**Affiliations:** ^1^Bryant Research, London, United Kingdom; ^2^Department of Psychology, University of Bath, Bath, United Kingdom; ^3^Department of Psychology, University of Zurich, Zurich, Switzerland; ^4^Department of Psychology, Lancaster University, Lancaster, United Kingdom

**Keywords:** animal product consumption, psychology, veganism, vegetarianism, meat

Animal production and consumption is at the root of many of the world's most pressing environmental, public health, and ethical issues. As well as contributing directly to greenhouse gas emissions, animal agriculture is incredibly resource-intensive and disruptive to ecosystems, driving water use, land use, biodiversity loss, and deforestation (1–3). Moreover, animal farms act as an incubator for emerging diseases, and a catalyst for antibiotic resistance (4, 5). Globally, over 90% of farmed animals are on factory farms, entailing small cage confinement, painful mutilations, and overall low welfare (6).

Increasingly, institutions including governments, public services, universities, and commercial food outlets are playing a role in reducing animal production and consumption (7). We have seen initiatives such as investments in alternative proteins (8), mandatory carbon and animal welfare labeling (9, 10), and nudges to encourage more sustainable food choices (11). While these institutions have an important role to play, they are ultimately beholden to individuals: generally, governments cannot implement policies without the support of voters, and companies cannot reshape their offerings without buy-in from consumers. Therefore, research into the public's attitudes about animal-product reduction and alternative proteins is a vital field of study.

This Research Topic called upon psychologists, behavioral scientists, and the broader scientific community to investigate the psychology of meat reduction, design and test interventions, and recommend ways forward to reduce the consumption of animal products. The resulting Research Topic contains over a dozen high-quality scientific studies covering a range of topics including vegetarian and vegan identity, moral psychology, behavior change, alternative proteins, health outcomes, and political science. All of these papers contribute to our understanding of relevant issues, which, in turn, can help to advance a more sustainable food system.

On vegetarian identity and moral psychology, behavioral scientists in Belgium provided an identity-based motivational account of resistance to veg^*^n advocacy. They theorized that veg^*^n (i.e., vegetarian and/or vegan) advocacy can threaten the moral and meat-eating identities of omnivores, which often causes them to engage in motivated reasoning to justify their consumption. They argue, however, that this apparent resistance often masks privately held beliefs that align with veg^*^n attitudes, and can precede later behavioral change (De Groeve et al.).

Scientists in Serbia found that individual and group affirmations of personally or collectively important values—for example, perceiving one's group as valuing democracy, trust, social connectedness, and solidarity in society—increased openness to meat reduction, including openness to cultured meat (Branković et al.). Likewise, psychologists in France showed how a perceived mismatch between an individual's own meat reduction and that of their group can motivate individuals to engage in behaviors (e.g., veg^*^n advocacy) aimed at positively shifting group norms (Harrington et al.).

Meanwhile, psychologists in the UK demonstrated a “halo effect” occurring for participants who were given positive environmental information about a cheese product. Relative to a no-environmental-information control condition, these individuals tended to infer that the environmentally-friendly product entailed higher animal welfare (their dairy cows treated better)—despite no information being given about the latter (Zamzow and Basso). As a result of this spreading positivity, they were more likely to endorse the product.

On behavioral change, psychologists in the Netherlands employed a reasoned action approach to investigate the attitudes that predict an intention to follow a vegetarian or vegan diet. They found that, in the Netherlands and the USA, instrumental and experiential attitudes (e.g., perceptions of dietary necessity and enjoyment, respectively) predicted dietary change intent. Additionally, in the Netherlands, descriptive norms about other people's intent to reduce their animal-product consumption predicted dietary change intent (Zaal et al.).

Economists in the Netherlands proposed that, with regard to animal products, dietary consumer groups can be modeled on a continuum. They investigated relative differences between meat abstainers, committed meat reducers, and avid meat eaters. They found that, compared to meat reducers, meat abstainers had more positive attitudes toward plant-based products and alternatives, such as tofu, veggie burgers, pulses and mushrooms. In comparison with avid meat eaters, committed meat reducers had a preference for non-meat animal proteins, such as eggs and cheese, and their diets were motivated more by environmental concerns and animal welfare (Verain and Dagevos). Compared to both other groups, avid meat eaters tended to be male and preferred to eat animal products over plant-based products.

Psychologists in Canada investigated the role of autonomous motivation—pursuing goals because one wants to, rather than has to—in maintaining a meat-free diet. In a longitudinal study of individuals transitioning to a veg^*^n diet, the researchers found no directional effect of autonomous motivation on dietary goal progress or goal facilitating behaviors. Nonetheless, goal progress within the study was related to subsequent reports of autonomous motivation suggesting that progress toward a veg^*^n diet may help build competence around meat-free eating (Kolbuszewska et al.).

A team led by psychologists in the UK followed Veganuary participants—meat eaters practicing a vegan diet for a month. They found that those who engaged with the pledge and reduced their meat consumption tended to develop stronger disgust reactions to meat afterwards, supporting the view that increased meat disgust follows (rather than precedes) meat reduction (Becker et al.). This demonstrates how meat avoidance—pursued through a pledge—can promote meat disgust and, possibly, spearhead future reduction.

On alternative proteins, marketing researchers in China demonstrated that increased intensity of social media marketing of plant-based meat can increase purchase intentions by influencing cognitive fluency, which broadens consumers' imaginations and reduces their perception of risk (Li et al.). Meanwhile, food and marketing scientists from across Europe investigated the impact of giving consumers health-related information for plant-based products, such as egg-free pasta, before or after tasting. They found that giving health information before tasting—rather than after or without—was associated with higher purchase intentions and stable taste perceptions across three phases of the experiment (Banovic et al.).

Further, a research team spanning Germany, the UK, and the USA investigated consumer perceptions of animal-free dairy from precision fermentation. In focus groups of potential early adopters from the United States, Germany, and Singapore, animal welfare considerations were among the most convincing, while concerns about consumer health, process safety and “messing with nature” were also shared. The researchers observed a cautious openness to animal-free dairy—an overall promising result for stakeholders investing in this emerging market (Broad et al.).

On political science, a researcher in the USA investigated the impact of emphasizing the environmental or animal rights case for meat reduction on simulated election performance. They found that, while the environmental case for meat reduction provoked a voter backlash, especially among Republicans, there was no such backlash for candidates who focused on farmed animal rights. In fact, candidates who demonstrated a personal concern for farmed animals received substantial boosts in voter support (Saha).

On health outcomes, medical researchers in China demonstrated that adherence to healthy plant-based diets was associated with better body composition in children aged 6–9. In particular, greater adherence was associated with lower abdominal obesity risk in girls, and stronger handgrip strength in boys (Chen et al.).

While environmental scientists continue to stress the importance of a shift away from industrially-farmed animal products, and food scientists develop ever-higher-quality alternatives, the social and psychological dimensions of consumer attitudes and behavior continue to present a challenge. All of the contributions in this Research Topic can help improve our understanding of the nexus of factors that impact on consumer decisions and individuals' choices and, ultimately, help to advance a more just and sustainable food system.

## Author contributions

All authors contributed to the writing of this editorial and the final version was agreed upon by all co-authors.
